# Draft Genome Sequence of a Highly Virulent Strain of the Plant Pathogen Dickeya solani, IFB0099

**DOI:** 10.1128/genomeA.00109-15

**Published:** 2015-03-19

**Authors:** M. Golanowska, M. Galardini, M. Bazzicalupo, N. Hugouvieux-Cotte-Pattat, A. Mengoni, M. Potrykus, M. Slawiak, E. Lojkowska

**Affiliations:** a Department of Biotechnology, Intercollegiate Faculty of Biotechnology, University of Gdansk and Medical University of Gdansk, Gdansk, Poland; b EMBL, EBI, Wellcome Trust Genome Campus, Cambridge, United Kingdom; c Department of Biology, University of Florence, Florence, Italy; d CNRS UMR5240 Microbiologie Adaptation et Pathogénie, Université de Lyon 1, INSA de Lyon, Villeurbanne, France

## Abstract

Dickeya solani is an important bacterial pathogen of potato cultivars in Europe. Here, we present the draft genome of D. solani strain IFB0099 isolated from potato in Poland that shows a high level of pectinolytic activity and a high virulence. This genome sequence is 5,094,121 bp and contains 4,365 protein-coding sequences.

## GENOME ANNOUNCEMENT

Bacteria belonging to the Dickeya genus in the *Enterobacteriaceae* family cause diseases on a wide range of plants (1), particularly on potato, chicory, banana, sunflower, and pineapple, and also on several ornamentals (*Dianthus, Dahlia, Chrysanthemum, Diffenbachia*, and Saintpaulia). Bacteria from the genus Dickeya cause maceration of plant tissue due to the degradation of the major plant cell wall components, pectin and cellulose (2). Highly virulent Dickeya strains have been recently isolated from potatoes (Solanum tuberosum) in several European countries (3, 4), and finally a new taxon, named Dickeya solani, was established (5). Strain IFB0099 (syn. IPO2276) was isolated in Poland in 2005 from a symptomatic potato plant (6, 7). Genetic analyses using repetitive sequence-based PCR (repPCR) and pulsed-field gel electrophoresis (PFGE) indicated high levels of homogeneity between D. solani strains isolated in different countries. However, IFB0099 was shown to be more virulent than the type strain of D. solani, IPO2222, isolated from a potato in the Netherlands in 2007 (8). Currently, the genome sequence of a few D. solani strains from various origins was sequenced (9–11). This could open the way to a comparative genomic analysis. Consequently, to evaluate genomic differences in depth, the sequencing of the IFB0099 genome was undertaken. The availability of the genome sequence of IFB0099 may be helpful in the elucidation/identification of the factors involved in its high virulence, in the development of biomarkers for D. solani detection, and for molecular epidemiology.

DNA was extracted with a NucleoSpin Tissue DNA extraction kit (MN, Germany), following the manufacturer’s instructions, and quantified by a Nanodrop spectrophotometer. Genome sequencing was performed by combining a 454 pyrosequencing method and PacBio SMRT technology to provide both high coverage and high-quality contigs (12). The 454 reads (191,539 reads) have been converted to the FastQ format using the seq_crumbs package (version 0.1.8; http://bioinf.comav.upv.es/seq_crumbs/) and trimmed using StreamingTrim (version 1.0) (13). Both 454 and PacBio reads quality has been checked using FastQC (version 0.10.1) (http://www.bioinformatics.babraham.ac.uk/projects/fastqc/). The PacBio reads (118,344 reads) coverage was found to be high enough to perform a hybrid assembly (~80×), using the pacBioToCA and the runCA program of the Celera assembler (12). The best assembly parameters have been checked using the Quast Web server (14).

The draft genome comprises 97 contigs. The total length is 5,094,121 bp, containing 4,365 protein-coding sequences (CDS) and 129 RNA-coding sequences detected after automatic annotation using prokka (version 1.7.2) (15). Among these, 88 tRNA sequences and 40 rRNA were found, corresponding to about 13 rRNA clusters. However, the number of rRNA clusters may be inferior, due to the draft status of the IFB0099 genome. The GC content is 56.4%. Pathogenicity-related genes, for example those coding for pectate lyases and cellulases, were annotated. A high level of homology was found with the best-characterized strain of the Dickeya genus, Dickeya dadantii 3937 (16).

### Nucleotide sequence accession numbers.

The draft genome sequence of D. solanii IFB0099 has been deposited in GenBank under the accession no. JXRS00000000. The version described in this paper is the first version, JXRS01000000.
